# A Nationwide Evaluation of Antibiotic Consumption in Kazakhstan from 2019 to 2023

**DOI:** 10.3390/antibiotics13121123

**Published:** 2024-11-23

**Authors:** Yuliya Semenova, Ademi Yergaliyeva, Ainur Aimurziyeva, Almira Manatova, Anargul Kuntuganova, Larissa Makalkina, Nurgul Aldiyarova, Daniil Semenov, Lisa Lim

**Affiliations:** 1 Department of Surgery, School of Medicine, Nazarbayev University, Astana 010000, Kazakhstan; anargul.kuntuganova@nu.edu.kz; 2 National Center of Public Healthcare, Astana 010000, Kazakhstan; ergalievaadema00@gmail.com; 3 School of Sciences and Humanities, Nazarbayev University, Astana 010000, Kazakhstan; ainur.aimurziyeva@nu.edu.kz; 4 National Scientific Center for Oncology and Transplantation, Astana 020000, Kazakhstan; m.almira@cancercenter.kz; 5 Department of Clinical Pharmacology, Astana Medical University, Astana 010000, Kazakhstan; makalkina.l@amu.kz; 6 Professional Association of Clinical Pharmacologists and Pharmacists, Astana 010000, Kazakhstan; aldiyarovan@mail.ru; 7 Computer Science and Engineering Program, Astana IT University, Astana 010000, Kazakhstan; daniil.semenov@nu.edu.kz; 8 Graduate School of Public Policy, Nazarbayev University, Astana 010000, Kazakhstan; lisa.lim@nu.edu.kz

**Keywords:** antimicrobial resistance, antibiotic consumption, antimicrobial stewardship, defined daily doses, Kazakhstan

## Abstract

Background/Objectives: There has been a lack of a holistic approach to evaluating antibiotic consumption in Kazakhstan over the past few years using an internationally recognized methodology. Therefore, this study aimed to provide a nationwide evaluation of antibiotic consumption in Kazakhstan during the period 2019–2023. Methods: Defined daily doses per 1000 inhabitants per day (DIDs) were calculated for systemic antibiotics (J01 code of the Anatomical Therapeutic Chemical Classification System (ATC)) following the methodology established by the Global Antimicrobial Resistance and Use Surveillance System (GLASS-AMC). The average annual percent change (AAPC) was computed for each chemical agent, pharmacological group, and J01 in general to evaluate past trends in antibiotic consumption. Results: The consumption of J01 antibiotics ranged between 10.869 DIDs (2022) and 14.470 DIDs (2020). There was an increase in antibiotic consumption during 2020 and 2021, but the overall trend was declining, with an AAPC of −2.45%. Azithromycin was the most consumed systemic antibiotic, followed by ceftriaxone and ciprofloxacin. The consumption of “Watch” group antibiotics prevailed in Kazakhstan during the study period, with the number of people consuming the top five “Watch” group antibiotics rising from 72,578 in 2019 to 94,617 in 2023. Conclusions: The findings of this study are crucial for the reorganization of the national antimicrobial stewardship program.

## 1. Introduction

Antimicrobial resistance (AMR) is often referred to as a “silent pandemic” and was listed by the World Health Organization (WHO) as one of the top 10 global public health threats [1]. AMR undermines the effectiveness of antibiotics and other antimicrobial agents, making it increasingly difficult to treat infections. It is estimated that 1.3 million people die from bacterial AMR every year, and this number is projected to increase to 10 million by 2050 [2]. Antimicrobial stewardship (AMS) programs are essential to combating AMR by promoting the judicious use of antimicrobial agents and improving patient outcomes. AMS aims to optimize the selection, dosage, and duration of treatments to minimize the development of resistance, reduce adverse effects, and lower healthcare costs [3].

AMR poses a particularly severe threat in low- and middle-income countries (LMICs), where the burden of bacterial infections is high, healthcare resources are limited, and antibiotic use is often poorly regulated [4]. In many LMICs, the lack of access to diagnostic tests and AMS strategies leads to the overprescription and misuse of antibiotics. This issue is further exacerbated by poor infection control practices, inadequate healthcare infrastructure, and the availability of over-the-counter (OTC) antibiotics, which facilitate the transmission of resistant bacteria [2]. Understanding the current patterns of antibiotic use and resistance in LMICs is important for developing targeted interventions and implementing AMS programs that are tailored to the specific challenges of these settings [5].

The Republic of Kazakhstan (hereafter referred to as “Kazakhstan”) is classified as an upper-middle-income country by the World Bank. The country gained its independence in 1991 following the dissolution of the Union of Soviet Socialist Republics (USSR). Its healthcare system follows the Semashko model and retains many features of the original model to this day [6]. Kazakhstan has a well-established network of hospitals that provide medical care to citizens free of charge. However, outpatient treatment is not reimbursed and is covered out-of-pocket [7]. Physicians are the only medical professionals authorized to prescribe pharmaceuticals, including antibiotics, and national standards of care are in place to guide prescriptions [6]. To address the emerging issue of AMR, the country has taken steps to implement AMS strategies by formulating a National Action Plan (NAP), establishing AMS programs in selected hospitals, and introducing legislation prohibiting the OTC sales of antibiotics [8]. Nevertheless, some pharmacies continue to sell antibiotics OTC [9].

Efforts have been made to analyze antibiotic consumption rates in community [9] and hospital sectors [10,11] using the “Access, Watch, Reserve” (AWaRe) classification [12]. Findings indicate that “Watch” group antibiotics predominated at both hospital and community levels, with cephalosporins being the most consumed pharmacological group. However, a holistic approach that evaluates antibiotic consumption across the entire healthcare sector is lacking, despite the need for such data to strengthen and prioritize AMS strategies. Thus, this study aimed to provide a nationwide evaluation of antibiotic consumption in Kazakhstan during the period 2019–2023.

## 2. Results

During 2019–2023, the consumption of antibiotics in Kazakhstan experienced a decline, with an average annual percent change (AAPC) of −2.45%. In 2020, antibiotic consumption peaked, reaching 14.470 defined daily doses per 1000 inhabitants per day (DIDs), followed by a decline in subsequent years. Among the antibiotics with increased consumption, colistin—a reserve group antibiotic—showed the most significant growth, with an AAPC of 148.16%, followed by tobramycin (AAPC = 123.83%) and cefoperazone combined with a beta-lactamase inhibitor (AAPC = 96.77%). The full list of antibiotics consumed in Kazakhstan during the period 2019–2023, along with their Anatomical Therapeutic Chemical (ATC) classification codes, pharmacological group, AWaRe category, DID, and AAPC, is presented in the Supplementary Materials (Table S1).

**Figure 1:**
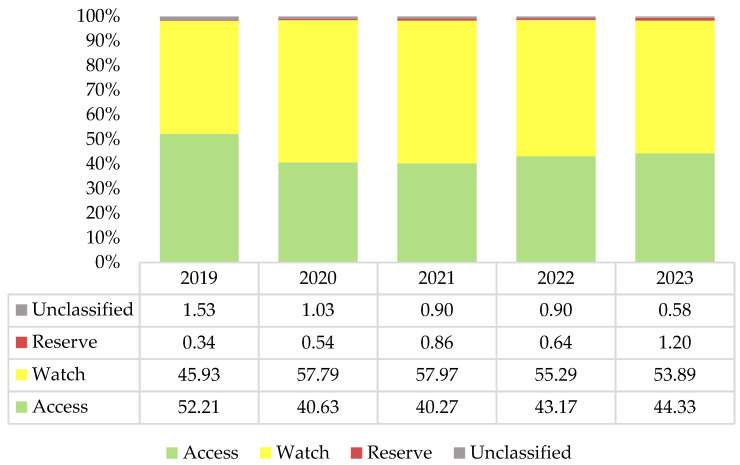
Antibiotic consumption by the category of the Access, Watch, and Reserve classification during 2019–2023, expressed as a percentage (%).

Figure 1 presents the dynamics of all antibiotics among the “Access”, “Watch”, and “Reserve” groups of the AWaRe classification. Overall, “Watch” group antibiotics constituted the majority of consumption, while the consumption of “Access” group antibiotics did not reach the 60% share recommended by the WHO. The share of “Reserve” group antibiotics gradually increased during the study period, reaching 1.20% in 2023. The “unclassified” group of antibiotics includes products that are unique to the region, and their share gradually declined from 1.53% in 2019 to 0.58% in 2023.

**Figure 2:**
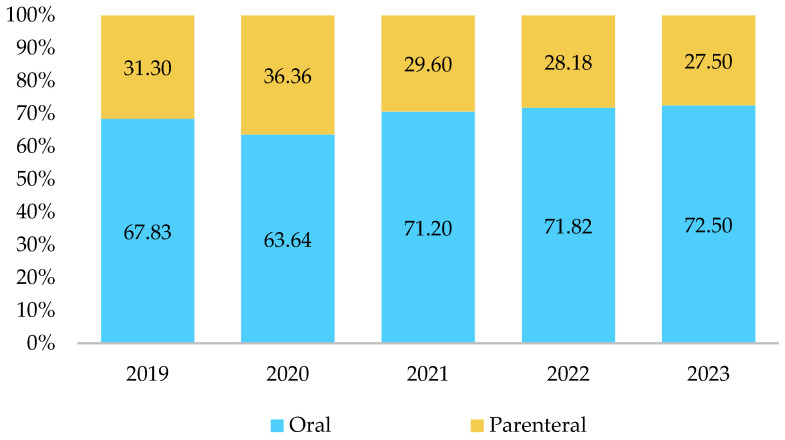
Antibiotic consumption by the route of administration, expressed as a percentage (%).

The oral route of antibiotic administration was predominant, gradually increasing in share and reaching 72.50% of total consumption in 2023. This trend was mirrored by a decline in the parenteral route, which only increased in 2020, reaching 36.36% of total consumption (Figure 2).

The consumption of tetracyclines, quinolones, macrolides, lincosamides, and streptogramins increased during the study period, with AAPCs of 1.0, 0.40, and 1.23, respectively. In 2020, the consumption of nearly all pharmacological groups of antibiotics rose, and the consumption of macrolides remained high in 2023, contributing to the large share of “Watch” group antibiotics. The share of other beta-lactams, including cephalosporins, also peaked in 2020 and has persisted, although their use tends to decrease gradually (Table 1).

Table 2 presents the top 90% most consumed antibiotics in Kazakhstan. Azithromycin was the most consumed antibiotic during the period 2019–2023. Its consumption increased in 2020 compared to 2019 (from 7.33% to 16.21%) and remained high through to the end of the observation period in 2023. Ceftriaxone and ciprofloxacin were the second and third most consumed antibiotics, respectively. These three “Watch” group antibiotics contributed significantly to the high share of “Watch” group antibiotics in overall consumption, accounting for 38.81% and 35.95% of the total consumption in 2020 and 2021, respectively. Interestingly, the most consumed “Access” group antibiotic, amoxicillin, was the most consumed antibiotic in 2019, but starting from 2020, it lost its position. Amoxicillin with clavulanic acid was consumed even less frequently than amoxicillin.

Table 3 presents the number of people in Kazakhstan consuming the top five antibiotics daily. Overall, the consumption of the top “Watch” group antibiotics peaked in 2020 (124,628) and showed an upward trend, with an AAPC of 4.40. Azithromycin had the highest consumption rate, with the number of users peaking in 2020 (43,985) and an overall AAPC of 7.42. Amoxicillin, the only antibiotic from the “Access” group among the top five, showed a decline in consumption, with an AAPC of −6.26.

## 3. Discussion

This study aimed to evaluate nationwide antibiotic consumption rates in Kazakhstan during the period 2019–2023. Overall, there was a declining trend in antibiotic consumption, with the AAPC equaling −2.45%. The most noticeable declines were observed for josamycin, kanamycin, and a combination of ampicillin with cloxacillin, which is not recommended by the WHO. The peak in antibiotic consumption occurred in 2020, when the difference compared to the preceding year (2019) nearly reached 3 DIDs. Although antibiotic consumption declined in 2021, it did not return to 2019 levels. Azithromycin, among other macrolides, contributed significantly to the observed increase. Since 2020, azithromycin has been the most consumed systemic antibiotic in Kazakhstan, which is alarming since it belongs to the “Watch” group of antibiotics. Overall, Kazakhstan remained within the “Watch” group zone, although a declining trend in the consumption of “Watch” group antibiotics has been observed over the past three years. However, the consumption of “Reserve” group antibiotics is growing, which is also alarming. The findings of this study need to be discussed to elucidate the factors contributing to these trends and to identify strategies for promoting more rational use of antibiotics in Kazakhstan.

Of interest is the comparison of the findings of this study with earlier reports on antibiotic consumption in Kazakhstan. The WHO report on the surveillance of antibiotic consumption indicated that the rate in 2015 was 17.89 DIDs [13], while the 2016 rate was reported to be 18.2 DIDs [14]. Although there are various studies investigating antibiotic consumption in community [9] and hospital sectors [10,11], to the best of our knowledge, this is the only study reporting the overall rate of antibiotic consumption for the most recent period. Another interesting observation is the decline in the share of antibiotics in the “Access” group compared to 2015 data reported by the WHO [13], while the share of “Watch” group antibiotics has increased, as evidenced by this study. However, the decreasing overall rate of antibiotic consumption is a positive finding and suggests that recent interventions and policies aimed at optimizing antibiotic use may be starting to show beneficial effects.

The consumption of amphenicols has significantly declined over the study period, largely due to a reduction in chloramphenicol use. This trend could be explained by the risk of bone marrow suppression associated with chloramphenicol intake, even though this side effect is relatively rare [15]. In addition, there was a marked decline in aminoglycoside consumption, primarily due to decreased use of kanamycin and gentamicin. This reduction could be attributed to concerns over their side effects, particularly ototoxicity, which typically results from prolonged use [16]. Similarly, the consumption of sulfonamides, particularly sulfaisodimidine, has significantly decreased. This may also be explained by longstanding concerns about associated adverse effects. These antibiotics have been available on the Kazakhstani pharmaceutical market for decades, so their side effects are well known to the medical community. Although newer classes of antibacterials also come with potential side effects, they are promoted by the pharmaceutical industry and have gained greater acceptance due to perceived benefits in efficacy, safety, and alignment with modern clinical practices [17].

There was an increase in antibiotic consumption during the COVID-19 pandemic, particularly in 2020 and 2021, when antibiotic consumption rates reached their highest levels during the study period, constituting 14.470 and 12.719 DIDs, respectively. Similar to many other countries around the globe, Kazakhstan experienced a dramatic shortage of healthcare resources, driven by elevated needs for both medicines and equipment [18]. Aside from the increased demand for healthcare resources, the COVID-19 pandemic also impacted the structure of medicine consumption, with antimicrobials being among the most affected due to their widespread, and sometimes inappropriate, use in managing COVID-19 and its secondary bacterial infections [19].

This study revealed that the consumption of azithromycin, a “Watch” group antibiotic, increased most dramatically, making it the leading antibiotic in terms of consumption starting in 2020. Azithromycin replaced amoxicillin, an “Access” group antibiotic, which was the most consumed antibiotic in 2019. The reasons behind this shift are worth investigating. Azithromycin was shown to have potential antiviral and anti-inflammatory effects, and early observations reported its anti-SARS-CoV-2 activity [20]. Nevertheless, later studies failed to confirm the benefits of azithromycin in treating COVID-19 patients [21]. Despite evidence disproving the usefulness of azithromycin in the treatment of COVID-19, the initial promising reports influenced clinical practice in Kazakhstan and continue to have an impact to this day.

The other two most consumed antibiotics in Kazakhstan were ciprofloxacin and ceftriaxone, both belonging to the “Watch” group. Over the study period, the consumption of ceftriaxone declined, possibly reflecting increasing resistance rates [22], while the consumption of ciprofloxacin remained largely unchanged and showed a slight increase. Since both of these antibiotics belong to the “Watch” group, there is a need to closely monitor their use and ensure adherence to AMS guidelines [23].

In comparison with other countries in the region, the consumption of antibiotics in Kazakhstan cannot be regarded as high. Among other Central Asian countries, antibiotic consumption in Tajikistan was 21.95 DIDs in 2015 [13], 25.7 DIDs in 2018 [14], and 16.6 DIDs in 2022 [24], while in Kyrgyzstan it was 17.94 DIDs [13], 20.5 DIDs [14], and 29.0 DIDs [24], respectively. In other countries of the former USSR, the consumption of antibiotics was even higher. For example, Georgia reported 24.44 DIDs in 2015 [13], 25.5 DIDs in 2018 [14], and 19.8 DIDs in 2022 [24], while Belarus reported 17.48 DIDs in 2015 [13], 19.0 DIDs in 2018 [14], and 16.3 DIDs in 2022 [24]. However, antibiotic consumption in some countries in the region was lower than in Kazakhstan. For instance, Uzbekistan reported 8.56 DIDs in 2015 [13] and 10.5 DIDs in 2018 [14], while Armenia reported 10.31 DIDs in 2015 [13], 10.7 DIDs in 2018 [14], and 9.6 DIDs in 2022 [24]. Kazakhstan could learn from these countries by analyzing their AMS programs to optimize antibiotic use and reduce overall consumption levels.

As for the countries of the European Union and the European Economic Area (EU/EEA), the population-weighted mean antibiotic consumption was 19.9 DIDs in 2019, 16.4 DIDs in 2020 and 2021, and 19.4 DIDs in 2022. Bulgaria, Cyprus, France, Greece, Poland, Ireland, Italy, Romania, and Spain were the countries where antibiotic consumption exceeded 20.0 DIDs during 2019–2022. Meanwhile, Austria and the Netherlands had antibiotic consumption levels of around 10.0 DIDs during the same period [25]. Different factors can be attributed to this variation in antibiotic consumption across EU/EEA countries, including differences in national healthcare policies, public awareness of AMR, the implementation of AMS programs, and variations in clinical practice guidelines. It should also be noted that Southern and Eastern European countries tend to have higher levels of antibiotic consumption, which may be influenced by cultural factors and prescribing behaviors [26].

In this study, the parenteral route of antibiotic administration ranged from 27.50% to 36.36% and showed a declining trend, which is a positive shift. However, the share of parenteral antibiotic administration in Kazakhstan remains higher than in other countries in the region. For example, in 2022, the parenteral route accounted for 21% of antibiotic administration in Georgia, 12% in Belarus and the Russian Federation, 13% in Armenia, and 15% in Azerbaijan. In Central Asian countries, the rates were higher, with 27% in Tajikistan and as high as 45% in Kyrgyzstan [24]. The predominance of parenteral administration is characteristic of the Soviet-style healthcare system, where many medications were administered parenterally, even in the community healthcare sector [6]. As several neighboring countries have made progress in reducing parenteral antibiotic use, Kazakhstan could benefit from adopting similar approaches and updating standards of care to promote the oral administration route both in community and hospital healthcare sectors.

This study has several strengths and limitations. The availability of a large nationwide dataset that combines antibiotic consumption data from both the community and hospital sectors is an obvious strength. However, this is also a limitation, as disaggregation by healthcare sector and by region within the country would provide more detailed insights. Another limitation is the relatively short study period of five years, which may not be sufficient to capture long-term trends in antibiotic consumption. In addition, this study only documented the rise in antibiotic consumption during the COVID-19 pandemic but did not analyze the possible contributing factors. Nevertheless, this is the most recent analysis of nationwide antibiotic consumption rates, which is essential for the reorganization of the AMS program currently being implemented in Kazakhstan.

## 4. Materials and Methods

This was a retrospective pharmacoepidemiological study on the consumption of antibacterials for systemic use (ATC code J01) in Kazakhstan. The study comprised three distinct stages: acquisition of consumption data, data cleaning and processing, and data analysis. The ATC/DDD Index 2024 was used to monitor J01 consumption over the past five years (2019–2023).

### 4.1. Data Acquisition

The data were sourced from the database created and maintained by Vi-ORTIS (Almaty, Kazakhstan), a pharmaceutical market research company [27]. The selection of this data provider was guided by the high quality of data collected, the meticulous approach to data validation, and the frequent use of Vi-ORTIS data in pharmacoepidemiological research [9,11].

Vi-ORTIS employs two different approaches for collecting data on community and hospital antibiotic consumption. Data on community consumption are collected through the “PharmCenter3.0” software, which is installed in approximately 75% of community pharmacies nationwide. This software is developed and maintained by Vi-ORTIS and provided to community pharmacies free of charge. Community consumption data account for both procurement of medicines by pharmacies and sales to patients, with a multi-level validation process that considers transfers of medicines between pharmacies and suppliers, checks returns, and includes other quality control measures. Data for pharmacies not covered by the “PharmCenter” software are modeled based on data from other pharmacies in the region [27].

Vi-ORTIS collects data on hospital consumption from “SK-Pharmacia”, the sole supplier of medicines to hospital facilities in Astana, Kazakhstan. “SK-Pharmacia” supplies both public and private hospitals and tracks supplies, returns, and transfers of medicines between the supplier and hospitals [28]. Therefore, the data obtained from Vi-ORTIS cover sales and supplies of antibiotics. To utilize these data for evaluating consumption, an assumption must be made that all sold/supplied antibiotics are consumed.

Vi-ORTIS updates its database on a monthly basis and provides access to third parties through a paid subscription, which enables the extraction of information on pharmaceutical substances, trade names, route of administration, dosage form, active ingredients per unit dose, the number of formulations per package, and the number of packages sold [27]. For the purposes of this study, the data were extracted on 31 January 2024 for the entire country for the period from 1 January 2019 to 31 December 2023.

### 4.2. Data Cleaning and Processing

The data extracted from the Vi-ORTIS database were entered into the Microsoft Excel template developed by the Global Antimicrobial Resistance and Use Surveillance System (GLASS-AMC). The GLASS-AMC is an international initiative under the WHO that aims to collect, analyze, and share data on antimicrobial consumption in participating countries. The Excel template developed by GLASS-AMC enables the evaluation of antibiotic consumption adjusted to the country’s population size [29]. This is achieved through the computation of the defined daily dose per 1000 inhabitants per day (DID) [30]. The data on antibiotic consumption for each year were entered into separate templates, and the demographic yearbooks issued by the Bureau of National Statistics were consulted to retrieve data on the population size of Kazakhstan for each respective year [31].

The Excel template developed by GLASS-AMC is arranged as a series of spreadsheets containing sections for data entry, information on ATC codes, formulas to calculate the DID and packages sold, and the desired level of disaggregation (e.g., healthcare sector, time period). The WHO developed a manual on data entry, which was referenced throughout the data entry process to ensure accuracy and consistency [29].

### 4.3. Data Analysis

Utilizing the GLASS-AMC template, the DID was calculated for each study year for the entire healthcare sector (community and hospital sectors combined) in disaggregation by using the ATC5 code. The calculated DIDs were then compiled into one Excel spreadsheet for further analysis, which was conducted using the Statistical Package for Social Sciences (SPSS) software, version 24.0. Given that the resulting data were in the form of a time series, the annual percent change approach was utilized to assess trends in antibiotic consumption over a period of 5 years (2019–2023) for each ATC5 code, pharmacological group, and systemic antibiotics in general [32]. The significance of the trend was determined using linear regression on log-transformed values, with *p*-values reported to assess statistical significance. Statistical significance for differences from zero was set at *p* < 0.05.

Based on the DID values, all antibiotics were ranked, and the top 90% most consumed antibiotics were identified and presented. The daily number of people consuming the top five antibiotics was calculated using the DID and the total population size [31], according to the following formula [32]:

Number of people consuming each antibiotic daily = (DID × population size)/1000

The WHO AWaRe classification was used to categorize all antibiotics into three main groups: “Access”, “Watch”, and “Reserve” [12]. Antibiotics with no known AWaRe group were categorized as “unclassified”.

## 5. Conclusions

The main positive finding of this study is the declining trend in total antibiotic consumption observed in Kazakhstan over the last five years, which can be attributed to the implementation of AMS strategies in the country. Nevertheless, this study has also highlighted concerning changes, the most significant of which is the large share of “Watch” group antibiotics, which constitute the bulk of antibiotic consumption, along with a growing trend in the use of “Reserve” group antibiotics. If this trend continues, it may lead to increased resistance and limited treatment options for infections, undermining the progress made by AMS efforts. To mitigate these risks, it is essential to enhance the surveillance of antibiotic use and resistance patterns while reinforcing AMS initiatives. Educational campaigns targeted at healthcare professionals and the public can raise awareness about the appropriate use of antibiotics. Continued monitoring and proactive management of antibiotic consumption are needed to control the potential rise in AMR.

## Figures and Tables

**Table 1:** Antibiotic consumption by pharmacological group, following Anatomical Therapeutic Classification level 3 (ATC3) codes.

Pharmacological Group	DID ^α^ (%)					AAPC ^0^ for DID *
	2019	2020	2021	2022	2023	
Tetracyclines (J01A)	0.565 (4.86)	0.593 (4.10)	0.570 (4.48)	0.505 (4.64)	0.644 (5.43)	1.00
Amphenicols (J01B)	0.322 (2.76)	0.287 (1.98)	0.285 (2.24)	0.249 (2.29)	0.268 (2.26)	−4.96 *
Beta-lactam antibacterials, penicillins (J01C)	2.169 (18.63)	1.848 (12.77)	1.836 (14.43)	1.741 (16.02)	2.185 (18.42)	−0.44
Other beta-lactam antibacterials (J01D)	2.598 (22.32)	3.822 (26.42)	3.036 (23.87)	2.500 (23.00)	2.752 (23.20)	−3.05
Sulfonamides and trimethoprim (J01E)	0.486 (4.17)	0.528 (3.65)	0.520 (4.09)	0.409 (3.76)	0.291 (2.45)	−12.03 *
Macrolides, lincosamides, and streptogramins (J01F)	1.453 (12.48)	2.886 (19.95)	2.453 (19.29)	1.903 (17.51)	1.902 (16.03)	1.23
Aminoglycoside antibacterials (J01G)	0.789 (6.78)	0.836 (5.78)	0.469 (3.68)	0.356 (3.28)	0.416 (3.51)	−19.22 *
Quinolone antibacterials (J01M)	2.161 (18.56)	2.575 (17.79)	2.546 (20.02)	2.237 (20.58)	2.366 (19.94)	0.40
Combinations of antibacterials (J01R)	0.030 (0.26)	0.023 (0.16)	0.003 (0.02)	0.000 (0.00)	0.000 (0.00)	-
Other antibacterials (J01X)	1.069 (9.19)	1.071 (7.40)	1.002 (7.88)	0.971 (8.93)	1.039 (8.76)	−1.54
Total	11.642 (100.00)	14.470 (100.00)	12.719 (100.00)	10.869 (100.00)	11.863 (100.00)	−2.45

^α^ DID—defined daily dose per 1000 inhabitants per day. ^0^ AAPC—average annual percent change. * *p* < 0.05 (for difference from zero).

**Table 2:** Top 90% most consumed antibiotics.

Substance	AWaRe * Category	Percentage by Year				
		2019	2020	2021	2022	2023
Amoxicillin	Access	10.91	7.98	6.57	13.71	8.88
Ceftriaxone	Watch	10.01	13.99	11.63	8.71	10.62
Ciprofloxacin	Watch	10.09	8.61	8.86	10.75	10.26
Cefazolin	Access	7.64	6.67	5.55	7.37	5.49
Azithromycin	Watch	7.33	16.21	15.46	6.77	11.82
Levofloxacin	Watch	6.25	7.11	8.79	6.44	7.66
Gentamicin	Access	6.01	5.13	2.87	6.22	3.09
Doxycycline	Access	4.21	3.54	3.59	3.98	4.22
Ampicillin	Access	3.83	1.61	2.70	3.84	2.34
Amoxicillin and beta-lactamase inhibitor	Access	3.50	2.93	4.88	2.94	6.87
Metronidazole	Access	3.10	2.80	2.32	2.76	2.89
Clarithromycin	Watch	2.77	2.17	2.55	2.75	3.13
Chloramphenicol	Access	2.76	1.98	2.24	2.68	2.26
Cefuroxime	Watch	2.66	3.67	4.26	2.65	4.06
Sulfamethoxazole and trimethoprim	Access	2.62	2.27	2.51	2.29	2.45
Nitrofurantoin	Access	2.38	1.44	2.30	2.28	2.29
Furazidin	Access	2.05	1.60	1.31	1.74	1.71
Sulfaisodimidine	Access	1.56	1.38	1.58	1.68	0.00
Nitroxoline	Unclassified	1.06	0.78	0.79	0.80	0.50

* AWaRe—Access, Watch, Reserve classification.

**Table 3:** Number of population receiving top 5 antibiotics every day.

Substance	AWaRe * Category	Number by Year					AAPC ^0^
		2019	2020	2021	2022	2023	
Azithromycin	Watch	15,803	43,985	37,354	29,260	27,714	7.42
Ceftriaxone	Watch	21,569	37,972	28,118	18,594	24,893	−4.18
Ciprofloxacin	Watch	21,741	23,364	21,405	22,950	24,059	1.86
Amoxicillin	Access	23,504	21,645	15,888	14,454	20,818	−6.26
Levofloxacin	Watch	13,465	19,307	21,255	15,735	17,951	3.77
Total for “Watch” group antibiotics		72,578	124,628	108,133	86,539	94,617	4.40
Total for Top 5 antibiotics		96,082	146,273	124,021	100,993	115,435	−0.03

^0^ AAPC—average annual percent change. * AWaRe—Access, Watch, Reserve classification.

## Data Availability

The data presented in this study are provided in the Supplementary Materials.

## Supplementary Materials

The following supporting information can be downloaded at: https://www.mdpi.com/article/10.3390/antibiotics13121123/s1, Table S1: Antibiotic consumption in community and hospital sectors combined, 2019–2023.
