# Supplementary material for: A Nationwide Evaluation of Antibiotic Consumption in Kazakhstan from 2019 to 2023
Source: Antibiotics (Basel). 2024 Nov 23;13(12):1123. doi: 10.3390/antibiotics13121123 (PMC11672648; doi:10.3390/antibiotics13121123)
Supplement: Supplementary file 1 [file antibiotics-13-01123-s001.zip › antibiotics-3283339-supplementary.pdf]

Table S1. Antibiotic consumption in community and hospital sectors combined, 2019-2023.

ATC5 code	Substance	Pharmacological group	AWaRe[∞] category	DID[α]					AAPC[0]
				2019	2020	2021	2022	2023	
J01AA02	Doxycycline	Tetracyclines	Access	0.490	0.512	0.457	0.417	0.501	-1.60
J01AA07	Tetracycline			0.076	0.081	0.113	0.087	0.144	14.55
J01BA01	Chloramphenicol	Amphenicols		0.322	0.287	0.285	0.249	0.268	-4.96*
J01CA01	Ampicillin	Penicillins		0.446	0.233	0.343	0.248	0.277	-8.48
J01CA04	Amoxicillin			1.270	1.154	0.836	0.700	1.053	-8.37
J01CE01	Benzylpenicillin			0.031	0.024	0.022	0.046	0.027	4.32
J01CE30	Combinations of penicillins		Unclassified[B]	0.014	0.011	0.005	0.008	0.007	-14.38
J01CR01	Ampicillin and beta-lactamase inhibitor	Beta-lactam	Access	0.000	0.000	0.000	0.000	0.003	-
J01CR02	Amoxicillin and beta-lactamase inhibitor			0.408	0.424	0.621	0.736	0.816	21.39*
J01CR05	Piperacillin and beta-lactamase inhibitor		Watch	0.001	0.001	0.003	0.001	0.001	12.81
J01CR50	Combinations of penicillins	Penicillins	Unclassified	0.000	0.002	0.006	0.002	0.000	-83.38
J01DB01	Cefalexin	First generation cephalosporins	Access	0.002	0.001	0.000	0.000	0.000	-
J01DB04	Cefazolin			0.889	0.965	0.707	0.676	0.651	-9.33*
J01DC02	Cefuroxime	Second generation cephalosporins	Watch	0.309	0.531	0.542	0.433	0.482	7.06
J01DC10	Cefprozil			0.018	0.013	0.011	0.015	0.025	7.47
J01DD01	Cefotaxime	Third generation cephalosporins		0.034	0.038	0.040	0.023	0.026	-10.04
J01DD02	Ceftazidime			0.043	0.059	0.052	0.042	0.042	3.72
J01DD04	Ceftriaxone			1.165	2.025	1.480	0.947	1.259	-5.86
J01DD08	Cefixime			0.075	0.058	0.074	0.087	0.126	15.29
J01DD12	Cefoperazone			0.001	0.002	0.001	0.001	0.000	-49.80
J01DD13	Cefpodoxime			0.027	0.046	0.055	0.048	0.051	14.12
J01DD15	Cefdinir			0.008	0.009	0.014	0.016	0.057	56.54*
J01DD62	Cefoperazone and beta-lactamase inhibitor		Unclassified	0.000	0.000	0.001	0.001	0.002	96.77
J01DE01	Cefepime	Fourth generation cephalosporins	Watch	0.009	0.026	0.019	0.189	0.010	23.57
J01DH02	Meropenem	Carbapenems		0.009	0.036	0.032	0.017	0.017	4.27
J01DH03	Ertapenem			0.004	0.005	0.003	0.003	0.001	-21.98*
J01DH04	Doripenem			0.002	0.005	0.004	0.003	0.001	-12.95
J01DH51	Imipenem and cilastatin			0.001	0.003	0.002	0.001	0.001	-10.67
J01EB01	Sulfaisodimidine	Sulfonamide-trimethoprim combinations	Access	0.181	0.200	0.201	0.120	0.000	-11.52
J01EE01	Sulfamethoxazole and trimethoprim			0.305	0.328	0.320	0.288	0.291	-2.19
J01FA01	Erythromycin	Macrolides	Watch	0.075	0.053	0.040	0.034	0.032	-19.14*
J01FA02	Spiramycin			0.071	0.061	0.055	0.028	0.054	-12.44
J01FA03	Midecamycin			0.033	0.034	0.027	0.020	0.010	-24.70*
J01FA06	Roxithromycin			0.022	0.015	0.010	0.009	0.009	-20.99*
J01FA07	Josamycin			0.050	0.040	0.005	0.000	0.000	-83.51
J01FA09	Clarithromycin			0.322	0.315	0.324	0.300	0.371	2.36
J01FA10	Azithromycin			0.854	2.345	1.966	1.490	1.402	5.54
J01FF01	Clindamycin	Lincosamides	Access	0.002	0.003	0.005	0.005	0.005	20.69*

J01FF02	Lincomycin		Watch	0.023	0.020	0.021	0.017	0.018	-6.53*
J01GA01	Streptomycin	Aminoglycosides	Watch	0.005	0.009	0.007	0.005	0.007	0.92
J01GB01	Tobramycin	Aminoglycosides	Watch	0.000	0.001	0.001	0.004	0.003	123.83
J01GB03	Gentamicin	Aminoglycosides	Access	0.699	0.742	0.365	0.319	0.367	19.22*
J01GB04	Kanamycin	Aminoglycosides	Watch	0.009	0.004	0.004	0.001	0.000	-54.66*
J01GB06	Amikacin	Aminoglycosides	Access	0.076	0.081	0.092	0.027	0.040	-21.34
J01MA01	Ofloxacin	Fluoroquinolons	Watch	0.086	0.116	0.112	0.086	0.118	3.45
J01MA02	Ciprofloxacin	Fluoroquinolons	Watch	1.175	1.246	1.127	1.169	1.217	0.08
J01MA03	Pefloxacin	Fluoroquinolons	Watch	0.002	0.000	0.000	0.000	0.000	-
J01MA06	Norfloxacin	Fluoroquinolons	Watch	0.080	0.081	0.095	0.087	0.056	-6.10
J01MA07	Lomefloxacin	Fluoroquinolons	Watch	0.002	0.002	0.001	0.002	0.002	1.71
J01MA12	Levofloxacin	Fluoroquinolons	Watch	0.727	1.029	1.119	0.801	0.908	1.95
J01MA14	Moxifloxacin	Fluoroquinolons	Watch	0.079	0.101	0.093	0.092	0.064	-4.91
J01MB04	Pipemidic acid	Quinolones	Unclassified	0.010	0.000	0.000	0.000	0.000	-
J01RA09	Ofloxacin and ornidazole		Unclassified	0.021	0.023	0.003	0.000	0.000	-
J01RA12	Ciprofloxacin and ornidazole		Unclassified	0.010	0.000	0.000	0.000	0.000	-
J01XA01	Vancomycin	Glycopeptides	Reserve	0.003	0.013	0.009	0.006	0.006	7.79
J01XB01	Colistin	Polymyxins	Reserve	0.000	0.000	0.001	0.001	0.001	148.16
J01XD01	Metronidazole	Imidazoles	Access	0.360	0.405	0.295	0.299	0.343	3.91
J01XD03	Ornidazole	Imidazoles	Access	0.001	0.000	0.001	0.001	0.001	2.23
J01XE01	Nitrofurantoin	Nitrofuran derivates	Access	0.277	0.208	0.293	0.291	0.271	2.96
J01XE03	Furazidin	Nitrofuran derivates	Access	0.239	0.232	0.167	0.183	0.202	-5.53
J01XX01	Fosfomycin	Phosphonics	Watch	0.021	0.021	0.027	0.034	0.015	-2.61
J01XX04	Spectinomycin	Imidazoles	Access	0.006	0.001	0.000	0.000	0.000	-
J01XX07	Nitroxoline		Unclassified	0.124	0.113	0.101	0.087	0.059	-15.93*
J01XX08	Linezolid	Oxazolidinones	Reserve	0.039	0.078	0.109	0.068	0.141	27.62
	Total DID**			11.642	14.470	12.719	10.869	11.863	-2.45

∞AWaRe – Access, Watch, Reserve Classification.

αDID – Defined Daily Doses per 1000 inhabitants per day.

⁰AAPC – Average Annual Percent Change.*p<0.05 (for difference from zero)
